# The role of vascular aging, bone marrow and immune system in hypertension

**DOI:** 10.1038/s41420-025-02851-9

**Published:** 2026-01-08

**Authors:** Yuwen Chen, Ming Yang, Wenhui Xie, Huashan Hong

**Affiliations:** Department of Geriatrics, Fujian Key Laboratory of Vascular Aging (Fujian Medical University), Fujian Institute of Geriatrics, Fujian Clinical Research Center for Senile Vascular Aging and Brain Aging, Fujian Medical University Union Hospital, Fuzhou, China

**Keywords:** Hypertension, Vascular diseases, Immunological disorders, Haematological diseases

## Abstract

Hypertension is a highly prevalent chronic disease all around the world, and the pathogenic mechanism is complicated. The early and rapid decline of the function of human vascular system due to the aging of human body are characteristics of hypertension, which is accompanied by progressive pathological remodeling and arterial stiffening. The pathogenetic action of oxidation and inflammation is the vital function in the process of endothelial dysfunction and arterial injury. Bone marrow is considered as the birthplace of the immune cell, and the role of bone marrow in hematopoiesis and immune response for the onset of hypertension has been confirmed. In turn, inflammatory and oxidative stress also affect the bone marrow and damage bone marrow function, causing a series of complications in hypertension, resulting in a vicious cycle. Recently, increasing evidence has suggested that bone marrow aging plays an important role in the onset and development of hypertension, and that the function of bone marrow in the pathogenesis of hypertension has been seriously overlooked. Bone marrow microvascular ageing is also involved in the progression of bone marrow ageing. Thus, this review mainly focuses on bone marrow function in aging and hypertension progression, addresses the current studies on the roles of vascular aging, the bone marrow and the immune system in hypertension, and discusses their interaction and function in the pathogenesis of hypertension. Furthermore, some novel molecular pathological mechanisms are surveyed. This can add a new impetus to the mechanism research of hypertension onset.

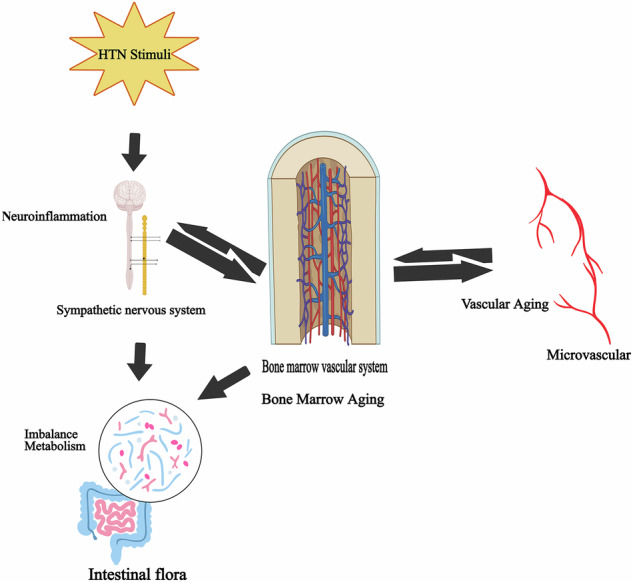

## Facts

- The acceleration and early occurrence of age-related decreases in vascular function are key features of hypertension.
- Activated immune system facilitates the progression of hypertension.
- Bone marrow aging plays a critical role in the pathophysiological mechanisms of hypertension.
- Vascular aging, especially microvascular aging in bone marrow promotes bone marrow aging via the immune system, thereby exacerbating hypertension.
- Several novel molecular mechanisms regulating these factors to accelerate the progression of hypertension have been elucidated and are further discussed.

## Open Questions

- How the interplay between bone marrow and vascular aging induces hypertension?
- What impact do bone marrow microvascular aging and bone marrow aging have on the progression of hypertension?
- What are the key molecular mechanisms involved in hypertension-linked Bone marrow and vascular senescence?

## Introduction

Cardiovascular disease is currently one of the most prevalent diseases in the world, with an unbroken decennial growing path almost worldwide. The total number of heart diseases patients increased by 252 million from 271 million in 1990, to 523 million in 2019 [1]. Patients with hypertension account for the largest proportion of reported cardiovascular disease. Hypertension patients present the largest number of registered cases of cardiovascular disease, and was the main driver of cardiovascular disease and premature deaths globally [2]. Recent estimates of its world-wide prevalence show that hypertension is on the increase resulting from age related-demographic change and a growing exposure to a set of modifiable risk factors: unhealthy diets characterized by excessive salt ingestion and low potassium consumption, combined with low physical activity [2, 3]. Besides, hypertension has been viewed as a condition of vascular aging relative to actual chronological age, which is characterized by vascular remodeling and stiffening, as well as endothelial dysfunction [4, 5]. Fibrosis, perivascular inflammation and vascular calcification are the key characteristics of vascular aging that induce vascular stiffening [6].

Degeneration associated with aging takes place in all organ systems as a typical physiological process. Within the vascular system, aging is marked by advancing pathological remodeling with the acceleration of stiffening. Early occurrence of vascular aging is documented in hypertension [7–11]. Factors that increase the likelihood of hypertension are strongly linked to a more rapid deterioration in vascular function. Thus, vascular age determination has become a significant part of cardiovascular disease prevention [10, 12].

Emerging and increasing data indicate a significant involvement of the activated immune system in the development of hypertension [13, 14]. The role of the immune system in hypertension is intricately linked to the sympathetic nervous system. The activation of sympathetic nerves aggravates immune inflammatory responses [15], and both central and peripheral inflammation can lead to excessive activation of sympathetic nerves [16]. While both are involved in the occurrence and development of hypertension, an increase in blood pressure has a positive feedback effect on central and peripheral inflammation [17–19], thus forming a vicious cycle.

Recent research has suggested that the bone marrow contributes to the development of hypertension in a way that was not previously recognized. As a source of immune cells, the hematopoietic function and immune responses of the bone marrow are associated with the pathogenesis of hypertension. Increased sympathetic drive to the bone marrow stimulates the release of pro-inflammatory progenitors that enter the brain and gut to promote inflammation [20]. Hypertension also affects the bone marrow, and increased sympathetic nerve firing accompanied by hypertension might activate and mobilize bone marrow cells with adrenergic receptors. Researchers need to further understand the dynamics of the bone marrow [21].

From a mechanistic perspective, aging induces the sustained presence of chronic low-grade inflammation within the immune system alongside oxidative stress. Additionally, with aging, blood vessels experience intimal thickening and calcification. Together, they can aggravate vascular endothelial dysfunction, microvascular ageing and target organ like bone marrow damage, which eventually lead to the occurrence and development of hypertension [22]. In the context of hypertension, overstimulation of the sympathetic nerve may prompt the bone marrow to initiate and sustain neuroinflammation, which in turn elevates blood pressure and establishes a detrimental feedback loop [23]. Studies have identified the role of SFRP5-MAPK14 and SFRP5-Wnt/β-catenin pathways, FKBP5/NF-κB axis, TGF-β1–NOX4 axis and S6K1-Arg2 axis and the function of Sirt1, Sirt4 and ANXA1 in the progress.

This review has linked vascular aging, bone marrow functions and the immune system to the pathology of hypertension. Several novel mechanisms regulating these factors to accelerate the progression of hypertension have been elucidated and are further discussed in the present review.

## Vascular aging and hypertension

Vascular aging, also known as vascular degenerative changes, refers to the physiological and pathological process of degeneration and aging of the functional structure of blood vessels under the action of many contributing factors, including smoking, obesity, elevated heart rate, blood pressure, blood lipids and other risk factors [24]. The progression of vascular aging underpins numerous vascular disorders and may influence the onset, development, and intensity of conditions associated with the vasculature [25]. It can manifest as a hemodynamic disorder, increased intima-media thickness, increased arterial stiffness, arteriosclerosis and plaque formation, vascular calcification, endothelial dysfunction, etc [26–30]. The pathogenesis of vascular aging is related to endoplasmic reticulum stress (ERS), autophagy, cellular senescence, mitochondrial dysfunction, DNA damage, telomere shortening, epigenetic alterations, oxidative stress, an aging-related secretory phenotype, and dysfunction of anti-aging gene silencing information regulator 1 [31, 32].

Vascular aging is evaluated mainly by morphology and function. The assessment of arterial structure is mainly non-invasive imaging, and the assessments include evaluating the integrity and function of the arterial lumen and wall, and identifying abnormal structure, vascular pulsation, and target organ perfusion [33, 34]. Alterations in vascular functionality occur prior to structural modifications, with the primary measures of functionality being arterial rigidity and endothelial performance. The vascular elasticity is weakened, the muscularis connection of the wall is broken, the elastic fibers are increased and arranged in a disorderly manner, and the collagen fibers are absent, further leading to structural changes as the endothelial function of the artery is damaged and the wall is hardened [35–38]. Serological alterations precede structural and functional modifications. Currently, molecular markers of vascular aging are focused mainly on studying cell aging, metabolism and inflammation [39, 40]. Persistent, mild inflammation is linked to age-related impairments in endothelial function and the development of atherosclerosis. Elevated concentrations of C-reactive protein (CRP), proinflammatory cytokines, and adhesion molecules, including tumor necrosis factor alpha (TNF-α), interleukin-6 (IL-6), and vascular cell adhesion molecule-1 (VCAM-1), serve as predictors of cardiovascular incidents [41–44]. Many other indirect biomarkers or indicators for vascular aging, such as endothelial progenitor cells, lymphocyte telomeres, advanced glycosylation end products also have good correlations with the direct measurement. To sum up, vascular aging is a multidimensional process, and a comprehensive set of parameters should be measured in clinical practice [45].

Vascular ageing is an active pathophysiologic cause and not consequence of hypertension in the elderlies. Thus, hypertension is one of the results of vascular aging [46]. The incidence rate of hypertension grows up with age, with more than 55% of elderly individuals over 65 years of age presenting with signs and symptoms [47]. Beyond age, many factors, including high consumption of sodium, low potassium, drinking alcohol, exposure to tobacco, being overwight, physical inactivity and diet, are also associated to a higher risk for having hypertension [2, 48]. At present, vascular age, a novel marker of health, is becoming popular for the diagnosis of a general impairment of heart, brain and kidneys and considered as an important independent risk factor of cardiovascular incidents and all-cause mortality in patients with hypertension [49, 50].

Interestingly, regarding human microcirculation, significant evidence has been gathered of different vascular territories, areas with vascular remodeling and endothelial disfunction in hypertensives [51]. Functional and structural abnormalities in microvascular are major determinants of the increase in mean blood pressure (BP) in primary hypertension and can lead to large artery stiffening, the high blood pressure can further induce functional and structural abnormalities of small artery [52]. This, in turn, highlights the importance of microvascular ageing for hypertension.

Genetic background may have significant effect in vascular aging and hypertension. Aortic vascular age index (AVAI) exhibited high genetic heritability, recent study has identified 54 independent genetic loci related to fibrillin-1 (*FBN1*) and elastin (*ELN1*) genes which is associated with AVAI, and the polygenic risk score for AVAI is closely related to the increasing risk of hypertension [53]. Mitochondrial DNA copy number (mtDNA CN) also has a complex relationship between vascular atherosclerosis and hypertension [54]. Besides, longevity-promoting resilience genes like *FLT1*, *Foxo3*, *GHR3*, *MAP3K5*, *PIK3R1* could mitigate mortality from hypertension, which further indicating the genetic influences in hypertension and vascular aging [55]. Integrating RNA-array datasets of senescent human coronary arterial endothelial cells (HCAECs) and aortic smooth muscle cells (HASMCs) as well as genome-wide association data for coronary artery disease (CAD) identified that *CST3* is functionally aided vascular aging, and COL4A1-ITGA1 and LPL-LRP1 pathways are related to the key processes of vascular aging and atherosclerosis development [56].

In summary, hypertension and vascular aging, as risk factors, are the basis for the development of many cerebrovascular diseases and act on different links in the cardiovascular event chain. Individuals with hypertension and advanced vascular aging experience a substantial rise in the likelihood of cardiovascular incidents. Genetic background has strong influences in both hypertension and vascular aging. Hypertension and vascular aging drive progression of each other, and improving hypertension and arteriosclerosis may help reduce the risk of adverse cardiovascular events in the clinic.

## Immune system dysregulation is a hallmark of hypertension

Hypertension is a chronic low-grade inflammatory disease. Research has consistently highlighted the essential function of the immune system in the development of hypertension and associated organ damage, as immune cells penetrating blood vessels, kidneys, fat tissue, and even the brain and other organs may alter the inflammatory milieu of tissues [57, 58]. Inflammatory dysregulation occurs during human hypertension. Vascular intima thickening, vascular calcification and changes in the body’s pro-inflammatory state result in an increase in a series of pro-inflammatory factors, which in turn cause persistent inflammation [59].

During the early stage of hypertension, inflammatory cells migrate to tissues and inflammatory factor expression will be induced and the body is kept in a chronic low grade inflammatory state. The renin-angiotensin-aldosterone system (RAAS) is subsequently activated, increasing blood pressure. The molecular signals of hypertension such as damage-associated molecular pattern (DAMPs), pathogen associated molecular patterns (PAMPs), activate Toll-like receptors(TLRs) and nucleotide-binding and oligomerization domain (NOD)-like receptor thermal protein domain associated protein 3 (NLRP3) inflammasome in pro-inflammatory macrophage(M1 macrophages) and DCs, respectively to drive the inflammatory process [59, 60]. Activating the immune system drives persistent mild inflammation and the corresponding oxidative stress and endothelial damage to affect the network of blood vessels and enhances the progression of hypertension [61]. It is also revealed that up-regulated TLRs would enhance inflammatory activity in hypertension. In addition, TLR4 antagonists decrease plasma concentration of norepinephrine levels and blood pressure in mice and also reduce oxidative stress and inflammatory responses in mice [62]. Thus, it shows important roles of the immune system and TLRs in hypertension pathology.

IL-12 has been shown to protect against Angiotensinogen II-induced hypertension, whereas IL-17 plays a role in enhancing pregnancy induced vascular and blood pressure abnormalities by activation and propagation of soluble natural killer cells, as evidenced by an increased expression in soluble natural killer cells by splenocytes from pregnant rats. Besides, the blood pressure is decreased in knockout mice in which mature lymphocytes have been removed by gene deletion. The above studies further confirmed that immune inflammatory factors, T cells and B cells play important roles in the occurrence, development and persistence of hypertension [63].

Hypertension can impair blood vessel and lead to inflammatory responses and vascular aging as well. Hypertension is closely associated with the activation of the immune system and causes the recruitment and accumulation of immune cells in organs that control blood pressure like the vascular system and kidney [64]. Cytokine release is also related to hypertension. It has been shown that various hypertensive stimuli may establish a network of multiple interleukins with various interactions [65]. In response of hypertension, IL-22 can act via the JAK2/STAT3 pathway leading to renal inflammation and fibrosis [66]. Infusion of IL-17 also has been investigated in many models of experimental hypertension as well as in patients [67]. Hypertensive patients also have elevated levels of IL-18, which acted on IL-18R and CD3 + T cells [68]. Further studies have shown that ANGII activates the PLC/IP3R/CA^2+^ pathway through its AT1R, triggering NLRP3 inflammasome assembly and caspase-1 activity, as well as an increase in IL-1β and IL-18 levels [69].

Overall, a considerable amount of data has demonstrated that the immune response and inflammation are involved in the pathogenesis of hypertension, hypertension can also lead to inflammatory responses and the crosstalk are closely related to target organ damage [22].

## Impact of aging bone marrow

The bone marrow is a key organ and main center for hematopoiesis, generating numerous immune cells essential for host defence and immune surveillance [70–74]. Aging bone marrow displays a series of cellular and molecular modifications, such as shift in the bone marrow stromal cell composition, increased adiposity, and dysregulation of signaling pathways [75, 76]. The accumulation of fat cells within the bone marrow is a critical characteristic feature of bone marrow aging process [77]. Adipogenesis could impair hematopoiesis and compromise immune cell production [71, 75, 78–80]. Disruption of signaling pathways, in the bone marrow, such as aberrant secretion of pro-inflammatory cytokines and alteration on niche factors expression also represent a key characteristic of aging bone marrow [81, 82], indicating bone marrow aging might contribute to systemic inflammation and the aging-associated phenotype.

The activity of hematopoietic stem cells (HSCs) in the ageing bone marrow wanes, based on reports [83–85]. Studies in human marrow show a change to an increasing myeloid and megakaryocytic cell fraction with age. It has been shown that lymphoid progenitors decrease with age in human marrow and that aged human HSCs demonstrate transcriptional programs of committed myeloid differentiation. In addition, phenotypic HSCs expand with age [86]. Clonal hematopoiesis is a marker of loss of heterogeneity in cells that contribute progeny to the peripheral blood [87]. Recently, mutations associated with clonal hematopoiesis are found often in elderly patients without a hematologic disorder [87–89]. Therefore, the “clonal hematopoiesis of indeterminate potential” (CHIP) is used in the case of this association because those mutations do not typically associate with hematologic disease in the elderly. As reported, 10-20% of patients over 70 years of age have CHIP [88, 90, 91]. Interestingly, recent studies, based on a murine model of atherosclerosis, have provided early evidence of a possible causal relationship between CHIP and vascular disease showing that CHIP correlates with increased cardiovascular risk [87, 91].

In all, the age-related alterations in the bone marrow microenvironment, including changes in HSCs function, alterations in immune cell production, and dysregulation of inflammatory signaling pathways, lead to immune dysfunction and abnormal hematopoiesis, which may contribute to age-related diseases such as hypertension [92].

## Bone marrow, immune system and hypertension

The role of the bone marrow in hypertension is incredibly untapped. Given the immune cell source site, the role of bone marrow in hypertension is tied with the immune response. Hypertension contains less circulating immune cells with potential for vascular healing and more bone marrow based pro-inflammatory immune cells present in gut and autonomic brains in the animal hypertension models [93–95]. Most importantly, there are more circulating gut-targeting pro-inflammatory immune cells in hypertensive individuals in comparison to blood pressure normal individuals [96]. The immune cells elevate the production of reactive oxygen species (ROS) and, therefore, contribute to oxidative stress. ROS has been recognized as pivotal pathogenic factor in the pathogenesis of hypertension [97]. It has been described that a higher cardio-oxidative stress is present in hypertensive patients [58, 98]. It has been shown that, in the progression of hypertension, oxidative damage was induced on bone marrow cells as well as disturbance with the hematopoiesis [99]. Oxidative stress in bone marrow cells is related to the activation of angiotensin II as well as intrinsic RAS. Angiotensin II induces NAD(P)H oxidase activation and the subsequent production of superoxide anions [100, 101]. It has been described in hypertensive mice that they have more lymphocytes and more ROS, meanwhile their undifferentiated bone marrow mononuclear cells (BM-MNCs) number is decreased [102]. In turn, monocytes originating from bone marrow can generate reactive ROS as well as secrete cytokines and matrix metalloproteinases [21], evidencing the connection between bone marrow and oxidative stress.

Inflammatory cells derived from the bone marrow of spontaneously hypertensive rats migrate to the brain, induces neuroinflammation and subsequently hyperactivates sympathetic nerves [93]. Also, co-culture of stellate neurons from both WT and spontaneously hypertensive rats with macrophages derived from bone marrow showed that macrophages from spontaneously hypertensive rats could amplify calcium currents in neurons that enhanced the responsiveness [103]. Additionally, deletion of monocytes/macrophages completely abolishes angiotensin II induced hypertension and to a great degree, corrects vascular function [104]. Studies have also shown that in spontaneously hypertensive rat model the number of inflammatory cells within bone marrow increases by more than 30% and endothelial progenitor cell count reduces to 50%. This is tightly correlated to increased sympathetic nerve activity in bone marrow. Impaired function of bone marrow worsens the inflammatory response and causes endothelial injury in hypertension [94]. Also, the sympathetic nerves within bone marrow are important for sustaining and guiding effector memory T cells. Studies show that the bone marrow provides a reservoir for memory T-cells and is a site where responses of T-cell activation are initiated [105]. Hypertension stimulation expands memory T cells in the bone marrow and is amplified by sympathetic nervous system activity [106]. Inhibition of β2 adrenergic receptors by drugs or gene editing can limit the survival and homing of effector memory T cells in the bone marrow [107]. Thus, modulating homing of immune cells to bone marrow and recruiting cells from bone marrow can be an area of future study.

**Fig. 1:**
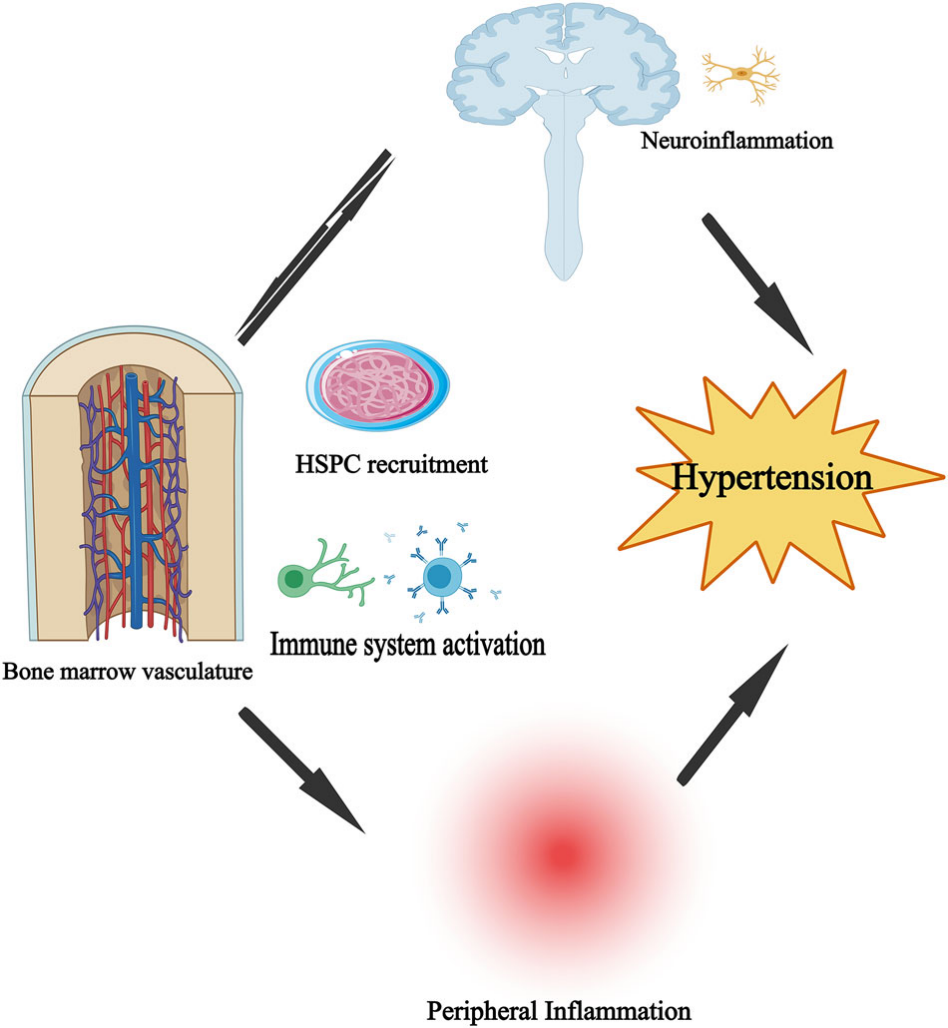
Role of bone marrow in hypertension. Neuroinflammatory effects from several stimuli act on the bone marrow. The bone marrow vasculature enhances the mobilization and discharge of hematopoietic stem cell progenitors (HSPC) and cytokines, thereby fostering neuroinflammation and peripheral inflammation, reinforcing the pro-inflammatory condition and playing a role in hypertension.

Taken together, these finding indicate that the bone marrow regulates innate and adaptive immunity, thereby participating in the development and progression of hypertension (Fig. 1).

## Immune system and bone marrow role in vascular aging

For vascular aging, greater evidence supports that bone marrow pathologies are linked to vascular lesions [108–110]. Cardiovascular disease involving reduced hematopoietic tissue, fat deposition, and decreased microvascular density, which leads bone marrow function [111], highlighting the role of microvascular function in BM. The bone marrow is the primary EPCs source and modulates hematopoiesis, via secreted and cell surface-bound signals received from HSCs [112–114]. It has been evidenced significant association between BM function and maintenance of endothelial cells [115, 116]. Endothelial cells form a barrier that regulates exchange with the blood pool and the trafficking of cells to and from the bone marrow [117]. As part of vascular walls, EPCs participate in many processes, including angiogenesis and regulation of blood pressure [118]. And vascular function controls numbers of circulating leukocytes, both at homeostatic and infectious levels [119–121]. The endothelial progenitor cells (EPCs) are required to repair the endothelial tissue damaged due to the inflammatory processes and other processes involved in the development of hypertension [122–124]. Inhibition of EPC’s reparative capacity could maintain the progression of vascular aging and pathogenesis of high BP [125–127]. Recent studies reveal that EPCs responded differently to specific stimuli. Short-term inflammatory stimulations mobilizes EPCs but, on the other hand, the long-term inflammatory response may also decrease the number of circulating EPCs [128]. Interestingly, stimulation of β-2 adrenergic receptors can enhance functionality of EPCs but persistent increase in norepinephrine (NE) in bone marrow could reduce functionality of EPCs [129]. All these indicate a correlation between vascular aging, hypertension, and bone marrow. Moreover, NE also functions as a vasoconstrictor in bone marrow and acts as a regulator of blood supply [130] Therefore, increased level of circulating NE can cause considerable vasoconstriction in bone marrow, forming a hypoxic niche that impacts functioning of the stem and progenitor cells, with consequent increase of the local inflammatory response [131].

Recent data from in vitro and clinical studies suggest that the immune system can affect endothelial dysfunction, leading to diabetic nephropathy, neuropathy and retinopathy associated with microvascular and macrovascular diseases like coronary artery disease, peripheral arterial disease, and stroke, which associated with old age [132, 133]. The immunosenescence-related disproportion in T lymphocytes may have significant impact on endothelial dysfunction, which is a key event in vascular aging [134]. Furthermore, the hypertension-associated endothelial dysfunction is characterized by an abnormal production of ROS, which subsequently reduces the vascular bioavailability of NO [51, 135, 136]. Recent studies have indicated that NOX-mediated oxidative stress significantly contributes to the impaired function of EPCs in numerous cardiovascular conditions, while suppressing NOX activity can enhance EPC functionality [137–139]. Besides, exercise-intervened cell-derived extracellular vesicles (cEVs) have the ability to protect cerebral microvascular endothelial cells against hypertensive and hypoxic injury [140], which link the bone marrow, immune system and vascular aging together. These ideas and connections deserve further study in the context of hypertension.

## Influence of bone marrow and vascular aging on hypertension

**Fig. 2:**
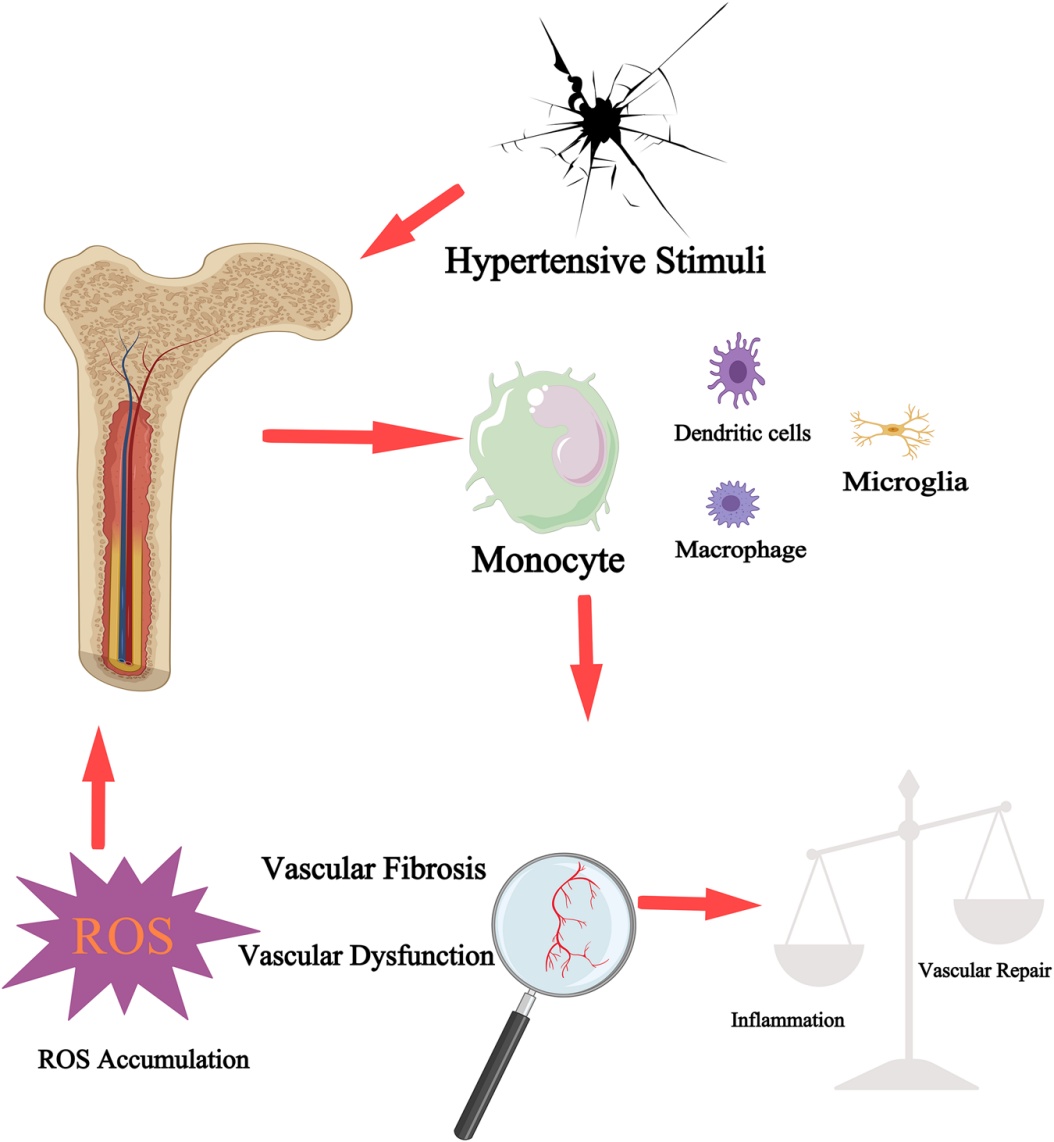
Interaction between hypertension and bone marrow. Various risk factors for hypertension stimulate the mobilization of monocytes and other inflammatory cells in the bone marrow, leading to increased inflammation and impaired bone marrow microvascular repair. The damage further affects the bone marrow to impair its function, thus facilitating the development of hypertension.

As mentioned above, studies have demonstrated a close relationship between bone marrow activity and endothelial maintenance, and aging bone marrow is associated with age-related anemia. Moreover, earlier research has likewise indicated a favorable correlation between hemoglobin levels and hypertension [141–143]. Reticulocyte levels are significantly positively correlated with hypertension in elderly individuals and significantly negatively correlated with atherosclerosis, which may help elucidate the background mechanisms of age-related endothelial activity, bone marrow activity and hypertension [144]. Abnormal hematopoiesis in the bone marrow could also advance cardiovascular disease by generating excess inflammatory leukocytes that attack the arteries and the heart. Bone marrow-derived monocytes and neutrophils accumulate in in the arterial wall and release pro-inflammatory cytokines to pivotally participate in the pathogenesis of atherosclerosis and hypertension [145–149]. On the other hand, hypertension remodels the vascular bone marrow niche, stimulating hematopoiesis and the production of inflammatory leukocytes. In the bone marrow of mice with hypertension, perivascular collagen deposition and integrin expression are upregulated. Besides, bone marrow vascular leakage is increased and vasodilatory responses to acetylcholine are impaired, suggesting endothelial dysfunction (Fig. 2). These findings further demonstrate the interaction between hypertension and the bone marrow vasculature [117].

The infusion of bone marrow cells not only attenuated arterial matrix remodeling, stiffening and hypertension but also improved humoral immune function which in turn reduced vascular inflammation, thus defining the importance of bone marrow in vascular aging and hypertension [150]. Furthermore, it was demonstrated the partial rejuvenation of bone marrow of old rats with cells of young rats promotes endothelial and vascular activation through up-regulation of microvascular density [151]. Bone marrow-derived cells (BMD- MSCs) deficient in suppressor of cytokine signaling 3 (SOCS3) conferred protection against systemic Ang II-induced vascular dysfunction, thus further establishing the importance of bone marrow in hypertension by providing new insight into the functional effects of SOCS3 in vascular and bone marrow compartments [152]. Bone marrow-derived mesenchymal stem cells (MSCs) also ameliorate the progression of vascular hypertrophy in a hypertension model by preventing the medial wall thickening, perivascular fibrosis, and vascular cell proliferation as well as the infiltration of innate and adaptive immune cells and their associated inflammatory cytokines. These findings depict the role of bone marrow in vascular remodeling [153] and also give new glimpses on the role of bone marrow in hypertension therapy.

## Signaling mechanism in hypertension-linked bone marrow and vascular senescence

Intimately understanding the molecular mechanisms of bone marrow and vascular aging is needed in order to devise new strategies to combat hypertension related bone marrow and vascular aging. Secreted Frizzled-related protein 5 (SFRP5) has been demonstrated to have an important role in the aging process. Plasma SFRP5 concentration positively correlates with aging and further studies has demonstrated that upregulating SFRP5 levels in aorta of old Sprague–Dawley rats protects against arterial inflammaging by inhibiting the inflammatory response, migration, and proliferation of arterial smooth muscle cells through the suppression of MAPK14 and Wnt/β-catenin signaling pathways [154]. Thus maybe Sfrp5 can affect bone marrow microvascular function through MAPK14 and Wnt/β-catenin signaling. The H_2_O_2_–NF-κB pathway contributes to the advancement of vascular aging as well. It has been found that NF-κB was activated in the carotid aorta of aged F344 rats, which enhanced the production of hydrogen peroxide (H_2_O_2_) [59]. The increased expression of FK506-binding protein 5 (FKBP5) is associated with NF-κB-induced activation of proinflammatory genes, potentially elevating cardiovascular risk. Both the genetic removal of FKBP5 and its pharmacological suppression can successfully counteract NF-κB-driven cardiovascular inflammation. Hence, targeting FKBP5/NF-κB signaling axis may be an effective therapy to retard inflammaging and improve microvascular health [155]. During aging, TGF-β1 in mouse vascular smooth muscle cells (VSMCs) significantly upregulates NOX4 levels, leading to transactivation of proinflammatory genes including VCAM1, IL6, CCL2,and CCL5, implying that TGF-β1–NOX4 axis underpins arterial inflammaging and underlining its potential for therapeutic targeting [156]. The ribosomal protein s6 kinase B1 (S6K1) plays a role in the aging process. Its function is intricately linked with that of arginase 2 (Arg2). S6K1-Arg2 axis can enhance endothelial nitric oxide synthase (eNOS/NOS3) uncoupling, elevated levels of intercellular adhesion molecules ICAM1 and VCAM1 and monocyte adhesion, thus leading to inflammation and senescence. Additional studies revealed that overexpression of S6k1 in cells that are not senescent leads to increased Arg2 expression, and the removal of Arg2 inhibits the S6K1-induced inflammaging by deactivating S6K1. This indicates the presence of a positive feedback mechanism linking S6K1 and Arg2 [157, 158]. Altogether, S6K1-Arg2 axis plays a crucial role in the onset and progression of endothelial senescence and hypertension associated bone marrow and vascular aging. In addition, Sirt1 also has been found to associated with arterial aging and endothelial dysfunction [159]. In aged mice, expression of Sirt1 is downregulated and activating Sirt1 in aged mice can boost endothelial function by upregulating cytochrome c oxidase COX2 (involved in endothelial dilation) protein levels. Besides, overexpression of Sirt1 can reduces arterial inflammation by deactivating aortic NF-κB and TNF-α and attenuate aortic production of superoxide via increasing antioxidant enzymes [160]. NF-κB/NLRP3 signaling pathway is a primary mechanism underlying vascular aging induced by bioaerosols in PM2.5 [161], which further highlight the role of NF-κB in vascular aging and hypertension. It has also been found that Sirt1 are downregulated in aged human aortic endothelial cells, whereas proinflammatory CCL2 protein is upregulated [162]. Sirt1 could therefore be a potential target for attenuating vascular inflammaging and aging. Belonging to the Sirtuins (Sirt) family as well, Sirt4 expression was upregulated following melatonin administration in rat fetal hypertension, suggesting that Sirt4 might be involved in mitigating the onset and progression of hypertension [163]. Also, flies with reduced Sirt4 expression have a shorter lifespan, while those with overexpressed Sirt4 live longer. The level of Sirt4 protein is decreased in the brains of aged rats as well [164, 165], which further indicated the role of Sirt4 in hypertension related aging. Recent research also identified NPRA/PKG/AMPK as a novel and critical signaling axis in the modulation of endothelial cell senescence, vascular aging and hypertension [166]. Dietary citrate improves mitochondrial function by activating activation of AMPK-related pathways, thereby delaying vascular aging and alleviating age-related vascular diseases [167]. Our previous study also implied that ANXA1 exerts protective effects of resolving inflammation and maintaining normal vascular homeostasis during the aging process via inhibiting TNF-α. which represents a novel aspect of vascular aging [168].

To sum up, SFRP5-MAPK14 and SFRP5-Wnt/β-catenin pathways, FKBP5/NF-κB axis, TGF-β1–NOX4 axis, S6K1-Arg2 axis NPRA/PKG/AMPK have been demonstrated to affect the progress of hypertension associated vascular ageing. In addition, the novel role of Sirt1, Sirt4 and ANXA1 in hypertension related bone marrow microvascular ageing deserves further study.

## Summary

Hypertension is the most common disease affecting humans, and presents significant cardiovascular and renal risks to patients [169]. It is a kind of age-associated diseases [170, 171] and age is a major risk factor for hypertension [172]. Age-related degenerative changes in arterial structure [37] and function promote the development of hypertension, and the development of hypertension accelerates the process of vascular aging, creating a vicious cycle [173]. Recent discoveries suggest that the aging process in any tissue, including vascular aging, is associated with a rise in inflammatory activity [174–176], underscoring the significance of inflammation and immune function in the development of vascular aging and hypertension. Growing evidence shows that chronic, low-grade inflammation in elderly individuals may contribute to the ongoing decline in cardiovascular function [177], and age-related arterial inflammation leads to endothelial cell senescence, thus promoting vascular aging [168].

Recently, studies have demonstrated the role of aging bone marrow in the progression of hypertension. The process of aging leads to a downregulation in bone marrow activity [178–180] Age-related changes in the bone marrow microenvironment, such as HSCs dysfunction, alterations in immune cell production, and dysregulation of inflammatory signaling pathways, are associated with various age-related diseases [91]. Studies have now proposed that oxidative stress may be a possible mechanism of vascular aging and has a function in the bone marrow [181] Endothelial senescence is expedited by oxidative stress, which compromises arterial function through a cooperative interaction with inflammation [37, 126]. This process is pivotal in vascular aging and disrupts bone marrow functionality. Immune cells involved in the progression of hypertension, such as monocytes, B cells and T cells, all originate in the bone marrow [182–184]. These bone marrow-derived cells accumulate in the perivascular space during hypertension and contribute to aortic stiffening [185]. In turn, inflammatory and oxidative stress also affect the bone marrow, causing damage to bone marrow function and leading to a series of complications in hypertension (Table 1).

**Table 1:** Effect of bone marrow in hypertension.

Reference	Finding	Methodology
[103]	1. Macrophages significantly augment the calcium responsiveness of sympathetic neurons. 2. Macrophages contribute to peripheral sympathetic overactivity during the initial phases of hypertension.	3–4 weeks male Wistar and spontaneously hypertensive rats (SHR) were used. The stellate ganglia were excised for cell culture
[94]	1. BM (bone marrow)-derived EPCs (endothelial progenitor cells) counts and function were decreased and BM-derived IC (inflammatory cells) counts and mobilization were increased. 2. Altered communication between the BM and the ANS correlates with abnormal BM function in cases of hypertension.	Twelve- to twenty-one-week-old adult male Wistar-Kyoto (WKY) and spontaneously hypertensive rats were utilized in the study.
[107]	1. Effector memory T cells gather in the bone marrow and may be reactivated through salt intake. 2. The formation and accumulation of effector memory T cells in the bone marrow is dependent on the presence of CD70 on antigen-presenting cells.	Wild-type mice, mice deficient in interferon-gamma (IFN-γ −/−) mice and mice deficient in CD70 (CD70 − /−), all on a C57Bl/6 J genetic background, were used in the work.
[150]	1. Accelerated senescence causes arteriosclerosis by impairing the humoral immune function in SAMP1 mice. 2. The BMC transplantation from miR-150-KO mice alleviated arterial matrix remodeling, stiffening, and hypertension in SAMP1 mice, partially through improving the humoral immune function to attenuated vascular inflammation.	The SAMP1 mice and miR-150 knockout mice (10months old) were used.
[151]	The partial revitalization of bone marrow in aged rats using cells from younger animals improves the recovery from ischemic damage.	Male SHR-SP rats, between 4 and 5 weeks of age, served as donors for bone marrow transplantation. Female SHR-SP rats, approximately 55 weeks old (55 ± 3 weeks) and kept on a high-salt diet for over 40 weeks, acted as recipients of the bone marrow cells.
[152]	Bone marrow-derived cells deficient in SOCS3 safeguard against systemic Ang II-induced vascular impairment.	Mice with a partial genetic deficiency in Socs3 (SOCS3 + /−) served as the model system.

*ERS* endoplasmic reticulum stress, *CPR* C-reactive protein, *TNF-α* tumor necrosis factor alpha, *IL-6* interleukin-6, *VCAM-1* vascular cell adhesion molecule-1, *RAAS* renin-angiotensin-aldosterone system, *DAMPs* damage-associated molecular patterns, *PAMPs* pathogen-associated molecular patterns, *TLRs* Toll-like receptors, *DCs* dendritic cells, *HSCs* hematopoietic stem cells, *CHIP* clonal hematopoiesis of indeterminate potential, *ROS* reactive oxygen species, *BM-MNCs* bone marrow mononuclear cells, *EPCs* endothelial progenitor cells, *SOCS3* suppressor of cytokine signaling 3, *MSCs* mesenchymal stem cells, *SFRP5* Secreted Frizzled-related protein 5, *FKBP5* FK506-binding protein 5, *VSMCs* vascular smooth muscle cells, *S6K1* Ribosomal protein s6 kinase B1.

**Fig. 3:**
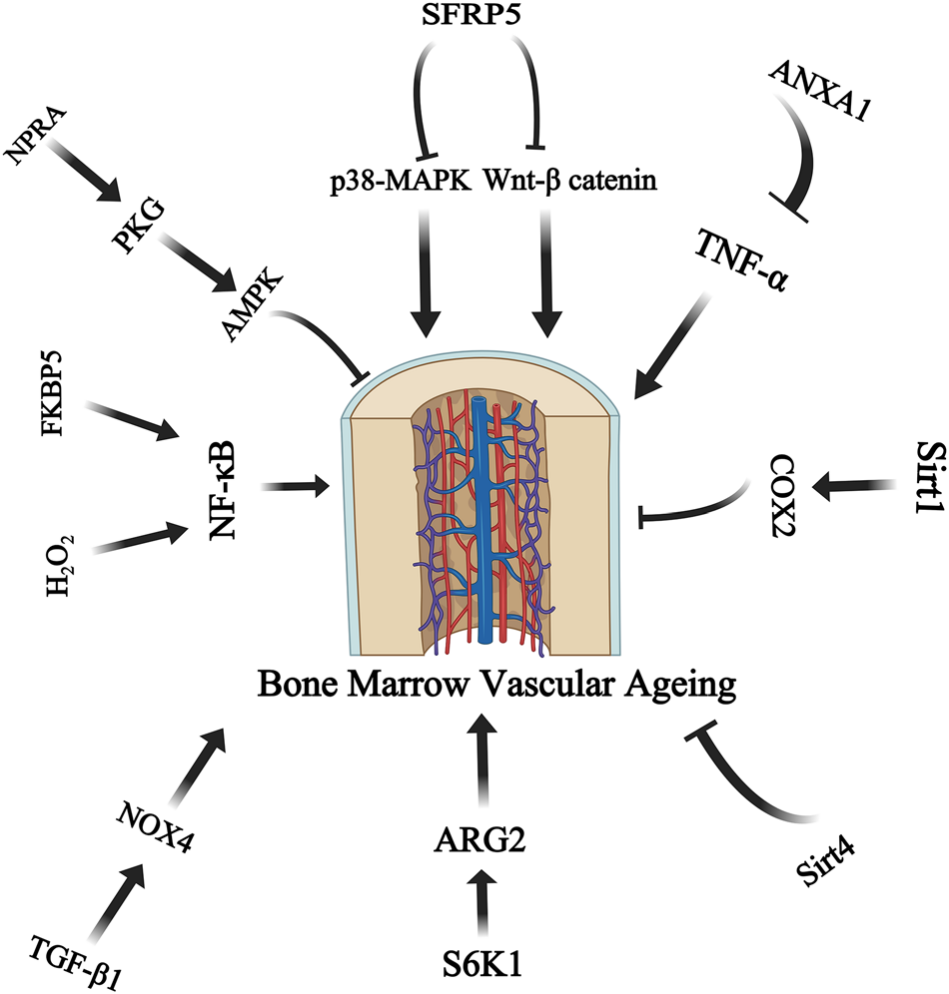
Underlying mechanisms of bone marrow microvascular ageing. Ageing triggers several molecular mechanisms that are conducive to the emergence of bone marrow microvascular disfunction.

To summarize, the aging process triggers persistent mild inflammation, oxidative stress, endothelial membrane thickening, and vascular calcification, all of which contribute to vascular aging and play a role in the onset and progression of hypertension. The progress can aggravate vascular endothelial dysfunction and target organ include bone marrow damage. Conversely, hypertension can also cause sympathetic hyperexcitation to induce bone marrow maintaining the inflammation, which further promote the rise of blood pressure and form a vicious circle. Thus, hypertension may be a bone marrow disease [21]. The function of bone marrow microvascular in hypertension may be associated with some classical signaling pathways, such as SFRP5-MAPK14 and SFRP5-Wnt/β-catenin pathways, FKBP5/NF-κB axis, TGF-β1–NOX4 axis and S6K1-Arg2 axis, previous studies also imply the role of Sirt1, Sirt4 and ANXA1 in the progress (Fig. 3). Research focusing on the specific molecular and signaling mechanisms of the interaction between bone marrow and the immune system may provide a novel concept for future antihypertensive therapies [58, 186–188].
